# Phospholipase Cε plays a crucial role in neutrophilic inflammation accompanying acute lung injury through augmentation of CXC chemokine production from alveolar epithelial cells

**DOI:** 10.1186/s12931-019-0975-4

**Published:** 2019-01-11

**Authors:** Kanoko Umezawa, Tatsuya Nagano, Kazuyuki Kobayashi, Ryota Dokuni, Masahiro Katsurada, Masatsugu Yamamoto, Yoko Yoshikawa, Tohru Kataoka, Yoshihiro Nishimura

**Affiliations:** 1 Division of Respiratory Medicine, Department of Internal Medicine, Kobe University Graduate School of Medicine, 7-5-1 Kusunoki-cho, Chuo-ku, Kobe, 650-0017 Japan; 2 Division of Molecular Biology, Department of Biochemistry and Molecular and Biology, Kobe University Graduate School of Medicine, 7-5-1 Kusunoki-cho, Chuo-ku, Kobe, 650-0017 Japan; 3 Kobe University Incubation Center, 1-5-6 Miyakojima Minami-cho, Chuo-ku, Kobe, 650-0047 Japan

**Keywords:** Phospholipase Cε, Acute lung injury, Acute respiratory distress syndrome, Alveolar epithelial cells, Inflammation, Mouse model, Neutrophil, Nuclear factor-κB, Cxcl5

## Abstract

**Background:**

We have shown that phospholipase Cε (PLCε), an effector of Ras and Rap1 small GTPases, plays pivotal roles in inflammation and inflammation-associated carcinogenesis by augmenting proinflammatory cytokine production from epithelial cells of various organs. The purpose of this study is to analyze its role in neutrophilic alveolar inflammation accompanying acute lung injury (ALI), focusing on that in alveolar epithelial cells (AECs), which are known to make a major contribution to the pathogenesis of ALI.

**Methods:**

We examine the effect of the *PLCε* genotypes on the development of ALI induced by intratracheal administration of lipopolysaccharide (LPS) to *PLCε* wild-type (*PLCε*^*+/+*^) and knockout (*PLCε*^*ΔX/ΔX*^) mice. Pathogenesis of ALI is analyzed by histological examination of lung inflammation and measurements of the levels of various cytokines, in particular neutrophil-attracting chemokines such as Cxcl5, by quantitative reverse transcription-polymerase chain reaction and immunostaining. Primary cultures of AECs, established from *PLCε*^*+/+*^ and *PLCε*^*ΔX/ΔX*^ mice, are used to analyze the roles of PLCε, protein kinase D (PKD) and nuclear factor-κB (NF-κB) in augmentation of LPS-induced Cxcl5 expression.

**Results:**

Compared to *PLCε*^*+/+*^ mice, *PLCε*^*ΔX/ΔX*^ mice exhibit marked alleviation of lung inflammation as shown by great reduction in lung wet/dry weight ratios, accumulation of inflammatory cells in the alveolar space and thickening of alveolar walls as well as the number of neutrophils and the protein concentration in bronchoalveolar lavage fluid. Also, LPS-induced expression of the CXC family of chemokines, in particular Cxcl5, is substantially diminished in the total lung and AECs of *PLCε*^*ΔX/ΔX*^ mice. Moreover, LPS-induced Cxcl5 expression in primary cultured AECs is markedly suppressed on the *PLCε*^*ΔX/ΔX*^ background (*p* < 0.05 versus *PLCε*^+/+^ AECs), which is accompanied by the reduction in phosphorylation of inhibitor κB (IκB), PKD and nuclear translocation of NF-κB p65. Also, it is suppressed by the treatment with inhibitors of PKD and IκB kinase, suggesting the involvement of the PLCε-PKD-IκB-NF-κB pathway.

**Conclusions:**

PLCε-mediated augmentation of the production of the CXC family of chemokines, in particular Cxcl5, in AECs plays a crucial role in neutrophilic alveolar inflammation accompanying ALI, suggesting that PLCε may be a potential molecular target for the treatment of acute respiratory distress syndrome.

**Electronic supplementary material:**

The online version of this article (10.1186/s12931-019-0975-4) contains supplementary material, which is available to authorized users.

## Background

Acute respiratory distress syndrome (ARDS) is a life threatening respiratory condition characterized by tachypnea, refractory hypoxemia and diffuse opacities on chest radiographs following sepsis, pneumonia and trauma [1]. The mortality of ARDS patients is around 35–40%, however, effective protocols for its treatment remains to be worked out despite exhaustive investigation [2]. The pathological development of ARDS is traditionally classified into 3 successive phases: exudative, proliferative and fibrotic [1]. The exudative phase is characterized by immune-cell-mediated damage to alveolar epithelial cells (AECs) and endothelial cells and by the dysfunction of the alveolar capillaries. In the proliferative phase, the numbers of abnormal type II alveolar cells and inflammatory cells are increased, and, in the fibrotic phase, fibroblasts infiltrate and replace cells in the alveoli and alveolar ducts as fibrosis develops. The current view of ARDS suggests that these 3 phases are overlapping with each other [3] . Classically, the most characteristic feature of ARDS is inflammation with heavy infiltration of neutrophils, which is mediated by elevation of proinflammatory cytokines such as interleukin (IL)-1β, IL-6, IL-8 and tumor necrosis factor-α (TNF-α). Additionally, leukocyte recruitment is orchestrated bychemokines, in particular the neutrophil-attracting chemokines, C-X-C motif ligands (CXCLs) and C-C motif ligands (CCLs) [1, 4]. The injured AECs act as the main source of these chemokines, thereby making a great contribution to the pathogenesis of ARDS [5]. In this study, we focuse on the role of Cxcl5, which is produced from AECs, different from myeloid cells which produce most chemokines and reported to play a key role in neutrophil activation during the pathogenesis of ARDS [6].

Phospholipase C (PLC) hydrolyzes phosphatidylinositol 4,5-bisphosphate to generate inositol 1,4,5-triphosphate and diacylglycerol (DAG), which function as vital intracellular second messengers through mobilization of Ca^2+^ from the intracellular stores and activation of DAG-target proteins such as protein kinases C and D (PKD), respectively [7]. Thirteen PLC isoforms are present in mammals and grouped into 6 classes: β, γ, δ, ε, ζ, and η, based on the similarities in their structures and regulatory mechanisms. Among them, PLCε is the only isoform regulated by small GTPases Ras and Rap [8], and further studies showed that it is also regulated by Rho small GTPases and heterotrimeric G protein Gα_12_, Gα_13_ and β_1_γ_2_ subunits [7]. Stimulation of G protein-coupled receptors (GPCRs) with their ligands such as lysophosphatidic acid (LPA), sphingosine-1-phosphate (S1P) and thrombin induces PLCε activation [9]. PLCε is expressed in structural cells such as epithelial cells and endothelial cells but not in immune cells such as granulocytes, lymphocytes and macrophages [10–12]. By utilizing genetically modified mice for *PLCε*, we demonstrated that PLCε plays a crucial role in experimentally induced inflammation; mice homozygous for the inactivated *PLCε*alleles (*PLCε*^*ΔX/ΔX*^ mice) exhibited markedly attenuated inflammatory responses in various animal models including the phorbor ester-induced dermatitis, hapten-induced contact dermatitis and dextran sulfate-induced colitis models [10, 11, 13] and transgenic mice overexpressing PLCε specifically in the skin keratinocytes spontaneously developed chronic dermatitis resembling human psoriasis [14]. Concurrently, *PLCε*^*ΔX/ΔX*^ mice showed marked resistance to tumor formation in the two-stage skin chemical carcinogenesis and the de novo intestinal carcinogenesis on the *APC*^*Min/+*^ background, which were associated with attenuation of cancer-associated inflammation [15, 16]. In the lung, we found that PLCε plays a crucial role in Th2-cell-mediated eosinophilic inflammation in the mouse model of ovalbumin-sensitized allergic bronchial asthma [12]. In this model, primary culture of bronchial epithelial cells was used to demonstrate that PLCε is required for TNF-α-induced pro-inflammatory cytokine production. Recently, we showed that, in human colon epithelial Caco2 cells, PLCε enhances pro-inflammatory cytokine expression through nuclear factor-κB (NF-κB) activation through activation of PKD, thereby recruiting and activating immune cells to initiate and sustain inflammation [13]. It was shown that the PLCε-PKD axis, activated by LPA receptor engagement, augments cytokine production via NF-κB activation through phosphorylation and degradation of inhibitor κB (IκB) by ribosomal S6 kinase (RSK), not by IκB kinase (IKK) in the canonical NF-κB pathway.

Thus, we examine the role of PLCε in AECs in the pathogenesis of experimentally induced ARDS by employing the mouse model of LPS-induced ALI.

## Methods

### Reagents

LPS from *Escherichia coli* O111:B4 (L2630, Sigma-Aldrich, St. Louis, MO, USA), IKK inhibitor BMS-345541 (401,480, Calbiochem, Darmstadt, Germany), PKD inhibitor CID755676 (476,495, Calbiochem) and proteasome inhibitor MG-132(Calbiochem) were commercially obtained. Primary antibodies against the following proteins were purchased from Cell Signaling Technology (Denver, MA, USA): β-actin (#4967), phospho-IκBα (Ser32) (14D4) (#2859), IκBα (#9242), phospho-PKD (Ser916) (#2051) and PKD (#2052). Other antibodies used were anti-CD45 (30-F11, eBioscience, San Diego, CA, USA), anti-CD32 (93, eBioscience), anti-Cxcl5 (PAA860Mu01, Cloud-Clone Corp, Katy, TX), anti-pan-cytokeratin (C-11, ab7753, Abcam, Cambridge, UK), anti-prosurfactant protein C (pro-SPC) (ab90716, Abcam), anti-podoplanin (bs-1048R, Bioss, Boston, MA, USA) and anti-NF-κB p65 (sc-109, Santa Cruz, Dallas, TX, USA). In addition, CF dye-labeled secondary antibodies, CF488A (20015) and CF555 (20231), were purchased from Biotium (Hayward, CA, USA).

### Animals

*PLCε*^*ΔX/ΔX*^ mice homozygous for the allele harboring an in-frame deletion of the catalytic X domain of PLCε and hence devoid of its lipase activity [15] were backcrossed to C57BL6JJcl mice (CLEA Japan, Tokyo, Japan) for at least 8 generations. All animals were maintained in the animal facility of Kobe University Graduate School of Medicine. The use and care of animals were reviewed and approved by the Institutional Animal Care and Use Committee of Kobe University (Permit Numbers: P130612, P130612-R1 and P130401).

### AEC culture

Primary culture of type II AECs was prepared from adult naïve *PLCε*^*+/+*^ and *PLCε*^*ΔX/ΔX*^ mice according to the previously described protocol [17–19]. Culture dishes (*Φ* = 10 cm) coated with anti-mouse CD45 antibody (40 μg) and anti-mouse CD32 antibody (15 μg) were used to get rid of leukocytes from the culture. The epithelial nature of the cultured cells was confirmed by positive pan-cytokeratin staining in at least 97% of the cells. They were further checked by positive staining for pro-SPC and podoplanin, specific markers of AECs (Additional file 1: Figure S1). On day 3, the cells were stimulated with LPS (500 ng/mL) for various times [6, 20] and used for further analyses. When necessary, the cells were treated with the IKK inhibitor (20 μM) for 30 min or with the PKD inhibitor (10 μg/ml) for 60 min before LPS stimulation.

### Quantitative reverse transcription-polymerase chain reaction (qRT-PCR)

Preparation of total cellular RNAs and qRT-PCR using them as a template were performed as described previously [12]. The primers used for the qRT-PCR analyses of various chemokine mRNAs are summarized in Additional file 2: Table S1. The relative mRNA levels were calculated according to the ΔΔCt method using the β-actin mRNA as an internal control.

### Western immunoblotting and immunofluorescence staining analyses for cytokines expressed in AECs

Cultured AECs were lysed in Cell Lysis Buffer (Cell Signaling Technology) and total cellular proteins (20 μg) were separated by sodium dodecyl sulfate-polyacrylamide gel electrophoresis (SDS-PAGE), followed by Western immunoblotting as described before [21]. Immunofluorescence staining was performed as previously described with minor modifications [22].

### Induction of ALI by LPS administration

*PLCε*^+/+^ and *PLCε*^ΔX/ΔX^ mice at 8–10 weeks of age were anesthetized by intraperitoneal injection of dexmedetomidine (40 μl; Maruishi Pharmaceutical, Osaka, Japan), midazolam (11 μl; Astellas Pharma, Tokyo, Japan) and butorphanol tartrate (13 μl; Meiji Seika Pharma, Tokyo, Japan). The mice were randomly divided into the ALI model group and the control group, and received intratracheal administration of 50 μl phosphate-buffered saline (PBS) with and without LPS (5 mg/kg), respectively [23]. Twenty-four hours later, the mice were sacrificed for dissection of the lungs or subjected to the collection of bronchoalveolar lavage fluid (BALF).

### Collection and analysis of BALF

BALF samples were collected by washing the lung three times with 0.8 ml PBS through a tracheal cannula placed into each mouse under anesthesia as described previously [12]. The number of leukocytes in the BALF was counted using a hemocytometer. The BALF was then centrifuged at 1500 × rpm for 10 min at 4 °C, and the cell pellet was suspended in 50 μl PBS, subjected to cytospin preparation and stained by Romanowski protocol using Diff-Quik (Sysmex, Kobe, Japan). At least 200 leukocytes on each slide were subjected to differential counting of macrophages, neutrophils, lymphocytes and eosinophils according to the standard morphological criteria.

### Protein and cytokine measurements in BALF

The concentration of total protein in BALF was measured by using the Bradford protein assay (Bio-Rad, Hercules, CA, USA). The concentration of Cxcl5 in BALF was determined using the ELISA kit (R&D systems, Minneapolis, MN, USA).

### Histopathologic and imunohistochemical analyses

The dissected lungs were filled with phosphate-buffered paraformaldehyde via intratracheal instillation and further fixed in phosphate-buffered paraformaldehyde. The lung tissues were embedded in paraffin, sectioned and subjected to staining with hematoxylin and eosin (H&E). The paraffin-embedded sections were also subjected to immunohistochemical staining with the anti-Cxcl5 antibody (15 μg/ml) and the anti-pro-SPC antibody (1 μg/ml) along with the peroxidase polymer anti-rabbit IgG reagents by using the Polymer method with ImmPRESS reagent (Vector Laboratories, Burlingame, CA, USA).

### Measurements of the lung wet/dry weight ratio

The dissected lung was immediately weighed to measure the wet weight. Subsequently, it was dried in an incubator at 60 °C for 24 h until its weight was stabilized and was subjected to the measurement of the dry weight. The wet/dry weight ratio was calculated by dividing the wet weight by the dry weight.

### Statistical analyses

Data are presented as the mean ± standard deviation (SD). Statistical analyses were performed using the Student’s unpaired *t*-test to compare the differences between two groups. If a *p-*value was smaller than 0.05, the difference was considered statistically significant.

## Results

### Alleviation of ALI in *PLCε*^*∆X/∆X*^ mice

**Fig. 1:**
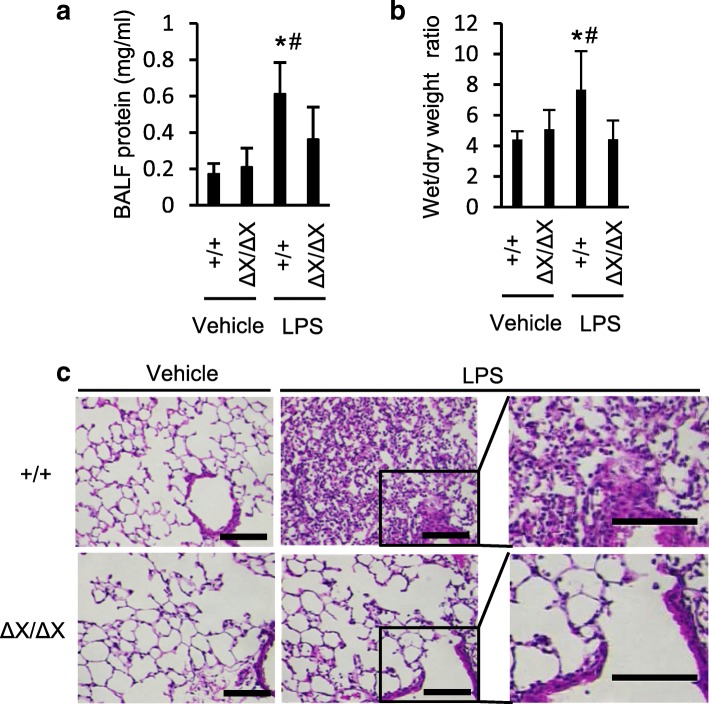
Effects of the *PLCε* genotypes on the development of LPS-induced ALI. **a** Effects on LPS-induced alteration of the alveolar capillary barrier . The total protein in BALF was measured. BALF was collected in 24 h after *i.t.* administration of LPS (5 mg/kg) or vehicle. *n* = 9 in the LPS-treated group, and *n* = 6 in the vehicle-treated group. * *p* < 0.05 between control and LPS administration, #, *p* < 0.05 between *PLCε* genotypes. **b** Lung edema was evaluated by determining the wet/dry ratios of the lung tissue isolated from mice in 24 h after *i.t.* administration of LPS or vehicle (*n* = 8 in the LPS-treated group and *n* = 4 in the vehicle-treated group). *, *p* < 0.05 between control and LPS administration, #, *p* < 0.05 between *PLCε* genotypes. **c** Histologic analysis of the lung. Lung sections were prepared in 24 h after administration of LPS or vehicle as in **a**, and they were stained with H&E. *n* = 3 in each group. Scale bar, 100 μm

To analyze the effect of the *PLCε* genetic background, the experimental model of LPS-induced ALI was applied to *PLCε*^+/+^ and *PLCε*^Δ*X/ΔX*^ mice. Twenty-four hours after the LPS administration, we examined the features characteristic of the experimental ALI. The increase in the protein concentration in BALF following the LPS administration, observed in *PLCε*^+/+^ mice, was attenuated in *PLCε*^*∆X/∆X*^ mice (*p* < 0.05, Fig. 1a). Concurrently, the LPS-induced increase in the lung wet/dry weight ratios, observed in *PLCε*^+/+^ mice, was greatly attenuated in *PLCε*^*∆X/∆X*^ mice (*p* < 0.05, Fig. 1b). These results indicated that both the increase in pulmonary permeability and the development of pulmonary edema were alleviated on the *PLCε*^*∆X/∆X*^ background. Histological analysis of the lung tissues revealed that *PLCε*^*∆X/∆X*^ mice exhibited great reduction in the LPS-induced accumulation of inflammatory cells in the alveolar space, which was indicative of attenuation of lung inflammation (Fig. 1c). Moreover, the LPS-induced thickening of alveolar walls, observed in *PLCε*^+/+^ mice, was substantially suppressed in *PLCε*^*∆X/∆X*^ mice, which was indicative of attenuation of the development of the alveolar capillary dysfunction.

**Fig. 2:**
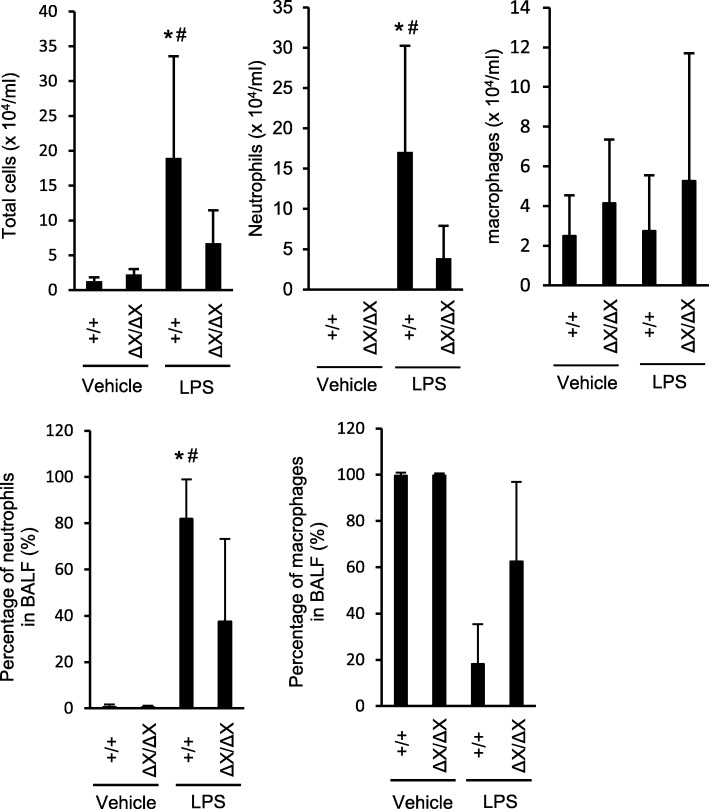
Effects of the *PLCε* genotypes on neutrophilic inflammation in LPS-induced ALI. BALF was collected in 24 h after *i.t.* administration of LPS or vehicle, cytospinned, and stained with a Romanowski stain (Diff-Quik). Leukocytes and neutrophils were counted under a microscope. *n* = 9 in the LPS-treated group, and *n* = 6 in the vehicle-treated group. *, *p* < 0.05 between control and LPS administration, #, *p* < 0.05 between *PLCε* genotypes

As neutrophils are known to play a crucial role in ALI, we examined the nature of the leukocytes infiltrated into the lungs by analyzing BALF collected in 24 h after the LPS administration. Differential leukocyte counting revealed that the LPS-induced increase in the total number of leukocytes in the BALF, observed in *PLCε*^+/+^ mice, was greatly suppressed in *PLCε*^*∆X/∆X*^ mice (*p* < 0.05, Fig. 2) and that the leukocytes were predominantly composed of neutrophils, whose number was also diminished in *PLCε*^*∆X/∆X*^ mice (*p* < 0.05, Fig. 2). The number of macrophages failed to show an increase depending on the LPS stimulation or the *PLCε*^+/+^ background (Fig. 2a). These results demonstrated that PLCε plays a crucial role in neutrophilic inflammation accompanying the LPS-induced experimental ALI.

### Crucial role of PLCε in chemokine production in AECs accompanying the LPS-induced ALI

**Fig. 3:**
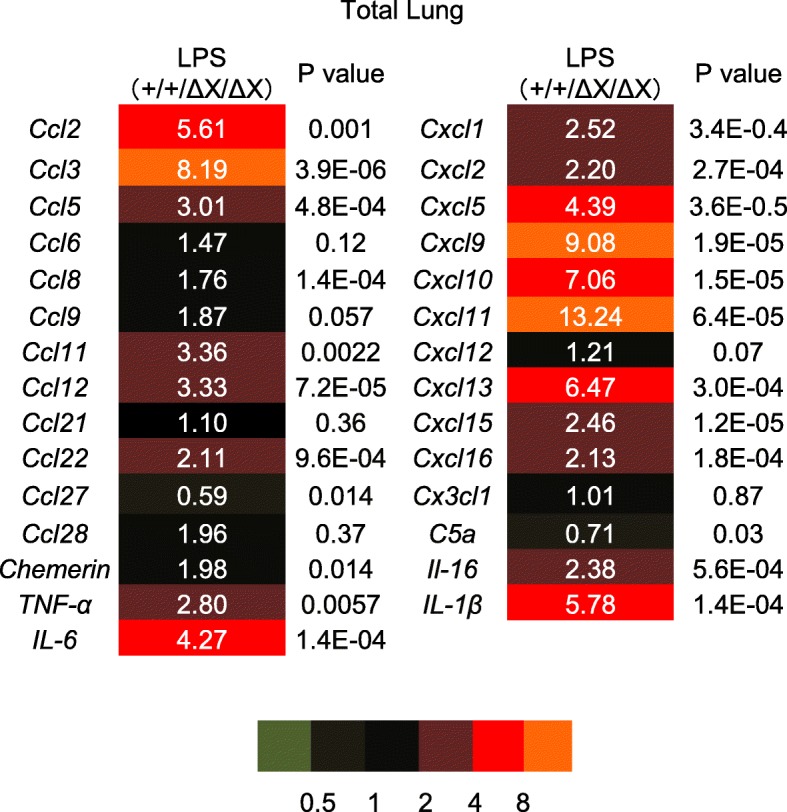
Role of gene expression of chemokines after LPS stimulation in mice lungs. Lungs from mice with the indicated *PLCε* genotype was collected in 24 h after *i.t.* administration of LPS or vehicle. The RNA was pooled from each group (*n* = 7 in the LPS-treated group, *n* = 4 in the vehicle-treated group) and subjected to qRT-PCR. Fold change was determined by dividing the relative mRNA level in *PLCε*^+/+^ mice with that in *PLCε*^∆X/∆X^ mice. Values represent *p-*values between *PLCε*^+/+^ and *PLCε*^∆X/∆X^ mice

**Fig. 4:**
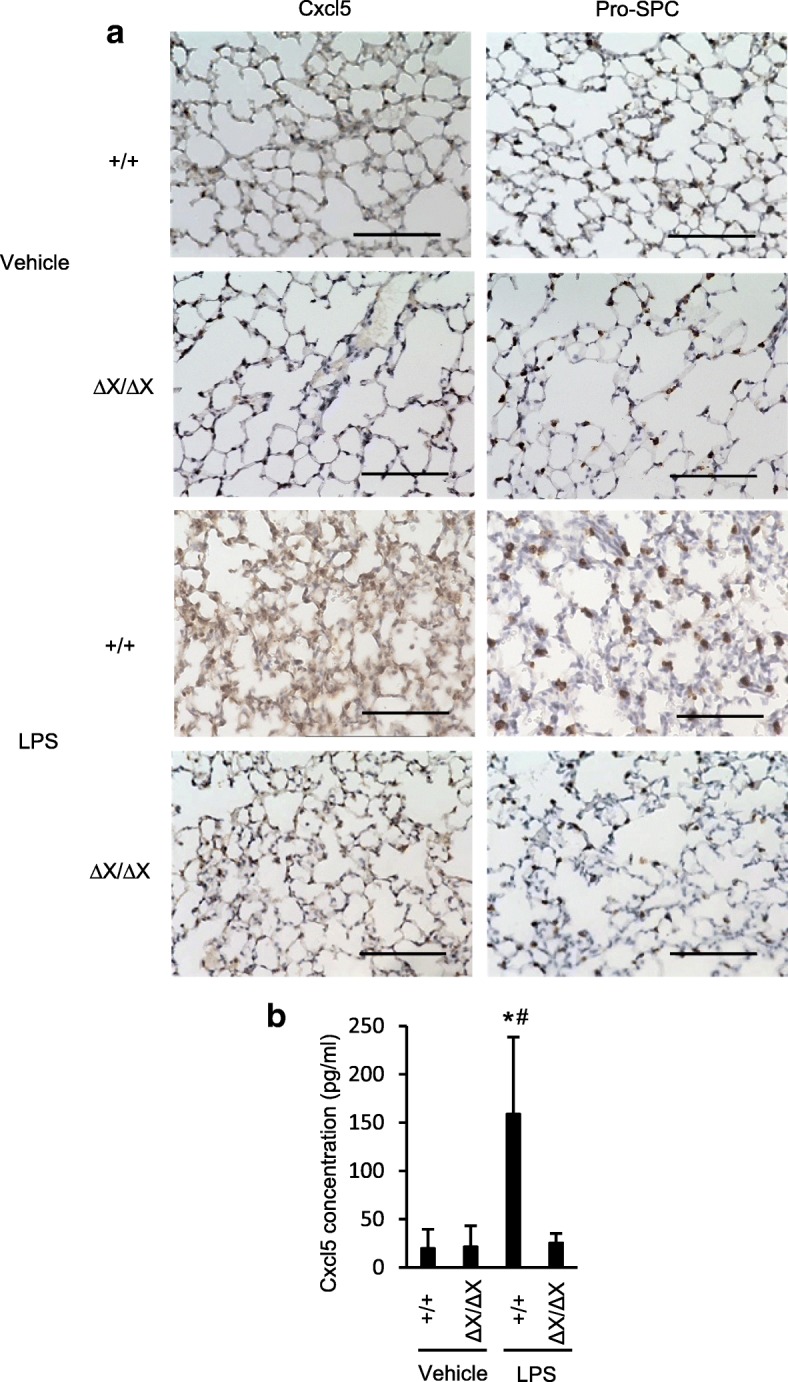
LPS-induced Cxcl5 expression in mice lungs. **a** Lung sections were prepared from both mice that were and those that were not subjected to LPS administration in the indicated *PLCε* genotype. Sections were subjected to Cxcl5 and pro-SPC immunostaining (*n* = 3 mice/group). Scale bar, 100 μm. **b** BALF was collected in 24 h after *i.t.* administration of LPS or vehicle, the expression levels of Cxcl5 in BALF were measured by ELISA. *n* = 9 in the LPS-treated group, and *n* = 6 in the vehicle-treated group. *, *p* < 0.05 between control and LPS administration, #, *p* < 0.05 between *PLCε* genotypes

We examined the mRNA levels of various proinflammatory cytokines in the whole lungs dissected from *PLCε*^+/+^ and *PLCε*^Δ*X/ΔX*^ mice in 24 h after the LPS administration by qRT-PCR. In the *PLCε*^+/+^ lungs, the expression levels of various cytokines, such as Ccl2/MCP1, Ccl3/MIP-1a, Ccl5/RANTES, Ccl9/MIP-1 g, Ccl11, Ccl12, Ccl22, Cxcl1/GRO-a, Cxcl2/MIP-2, Cxcl5/LIX/GCP-2, Cxcl9/MIG, Cxcl10/IP-10, Cxcl11/I-TAC, Cxcl13/BLC/BCA-1,Cxcl15/Lungkine, IL-1β, IL-6 and TNF-α, showed marked elevation following the LPS administration (Fig. 3 and Additional file 1: Figure S2). Among them, the LPS-induced expression levels of Ccl2, Ccl3, Ccl5, Ccl11, Ccl12, Ccl22, Cxcl1, Cxcl2, Cxcl5, Cxcl9, Cxcl10, Cxcl11, Cxcl 13,Cxcl15, IL-1β, IL-6 and TNF-α showed substantial reduction in *PLCε*^Δ*X/ΔX*^ mice (*p* < 0.05, Fig. 3 and Additional file 1: Figure S2). Intriguingly, they contained Cxcl1, Cxcl2, Cxcl5 and Cxcl15, which function as the chemo-attractants for neutrophils [24]. Among them, we focused on Cxcl5 because it was known to be produced mainly by AECs and induce neutrophil trafficking in response to LPS exposure in the lung [24]. To clarify the nature of the cells expressing Cxcl5, we carried out immunohistochemical staining of the lung sections prepared from *PLCε*^+/+^ and *PLCε*^Δ*X/ΔX*^ mice in 24 h after the LPS administration. The result showed that the LPS-induced increase in the Cxcl5 levels was observed mainly in the AECs, which was substantially attenuated in *PLCε*^∆X/∆X^ mice (Fig. 4a). The concentration of Cxcl5 in BALF also exhibited a great increase following the LPS administration in *PLCε*^+/+^ mice, which was almost completely suppressed in *PLCε*^Δ*X/ΔX*^ mice (Fig. 4b). These findings suggested an augmenting role of PLCε in the production of Cxcl5 in the exudative phase.

### Crucial role of PLCε in chemokine production in cultured AECs

**Fig. 5:**
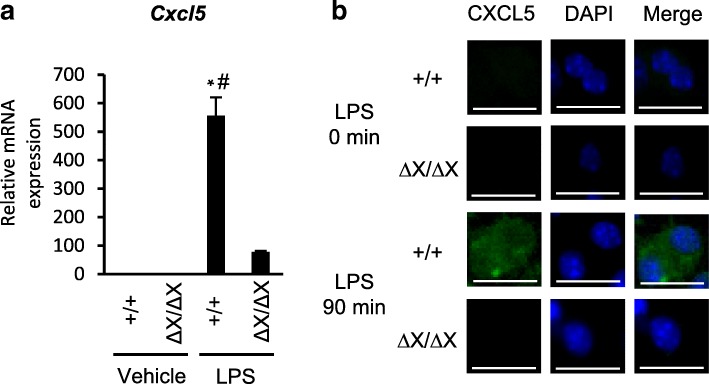
Role of PLCε in LPS-induced chemokine production. **a** The RNA was collected from the primary cultures of *PLCε*^+/+^ and *PLCε*^∆X/∆X^ AECs in 16 h after the administration of LPS (500 ng/mL) or vehicle (*n* = 8 mice/group). The RNAs were pooled from each group and subjected to qRT-PCR. *, *p* < 0.05 between control and LPS administration, #, *p* < 0.05 between *PLCε* genotypes. **b** Primary cultured lung AECs from *PLCε*^+/+^ and *PLCε*^∆X/∆X^ mice were prepared for Cxcl5 (green) immunostaining after stimulation for 90 min by LPS (500 ng/ml) (*n* = 8 mice/group). The Cxcl5 antibody (15 μg/ml) was used. Nuclei were counter-stained with 4′,6-diamidino-2-phenylindole (DAPI) (blue). Scale bar, 25 μm

We next examined the role of PLCε in LPS-induced Cxcl5 expression by using primary cultures of AECs. The primary cultured AECs established from *PLCε*^+/+^ mice exhibited a marked increase in the *Cxcl5* mRNA levels in 16 h after LPS stimulation, which was largely suppressed in those established from *PLCε*^Δ*X/ΔX*^ mice (Fig. 5a). Also, mmunofluorescence staining was used to confirm the LPS-induced Cxcl5 expression in *PLCε*^+/+^ AECs and its suppression in *PLCε*^∆X/∆X^ AECs (Fig. 5b). Thus, the PLCε-dependent augmentation of the LPS-induced Cxcl5 expression was recapitulated in vitro. Concurrently, the expression of Cxcl1, Cxcl2 and Cxcl15, Ccl2, Ccl3, Ccl5, Ccl11, Ccl21 and Ccl27 was elevated in a manner similar to that of Cxcl5 (Additional file 1: Figure S3A, B). By contrast, the elevated expression of Cxcl10, Cxcl11, Ccl12, Ccl22, TNF-α, IL-1β and IL-6 depending on the LPS stimulation and the *PLCε*^+/+^ background, occurred in the whole lung (Fig. 3 and Additional file 1: Figure S2), was not observed in AECs. These differences might be accounted for by the contribution of cells different from AECs, such as infiltrated immune cells present in the lung.

### Crucial role of the PLCε-PKD-NF-κB signaling in LPS-induced Cxcl5 expression

**Fig. 6:**
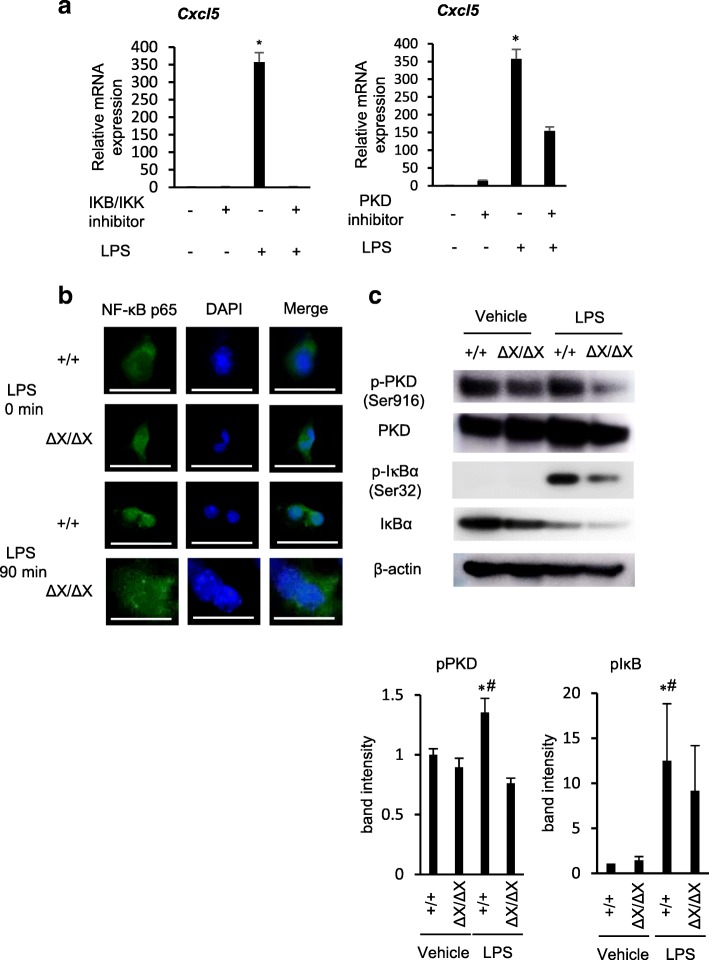
Role of PLCε in LPS-induced NF-κB activation. **a** Primary-cultured AECs from *PLCε*^+/+^ mice were treated with and without the IKB/IKK inhibitor (20 μM) or the PKD inhibitor (10 μg/ml) 0.5 h and 1 h before administration of LPS (500 ng/mL) or vehicle for 16 h. Expression of the *Cxcl5* gene was analyzed by using qRT-PCR (*n* = 7/group). Values represent *p-*values between LPS-stimulated AECs and LPS-stimulated AECs with the IKB/IKK inhibitor or the PKD inhibitor. *, *p* < 0.05. **b** Primary cultured AECs from *PLCε*^+/+^ and *PLCε*^∆X/∆X^ mice were subjected to NF-κB p65 (*green*) immunostaining after stimulation for 90 min with LPS (500 ng/ml) (*n* = 8 mice/group). The anti-NF-κB p65 antibody (1:50) was used. Nuclei were counter-stained with DAPI (*blue*). Scale bar, 25 μm. **c** Proteins, prepared from the primary cultures of *PLCε*^+/+^ and *PLCε*^∆X/∆X^ lung AECs in 1.5 h after treatment with LPS (500 ng/ml) or vehicle (*n* = 8 mice/group) were subjected to Western blotting with the indicated antibodies (*upper panel*). The intensity of each phospho-protein band was divided by the intensity of each total protein band. The average intensities obtained from at least three independent experiments were shown as the mean ± S. D with *p*-values (*lower panels*). *, *p* < 0.05 between control and LPS administration, #, *p* < 0.05 between *PLCε* genotypes

LPS is known to induce NF-κB activation through engagement of TLR4 via the canonical pathway, where the phosphorylation of IκB by IKK causes ubiquitination and subsequent proteasome degradation of IκB, thereby allowing NF-κB, a dimer of p65 RelA and p50, to undergo nuclear translocation and activate the expression of the target genes including those of various chemokines in an early time course [25]. We showed that, in human colon epithelial Caco2 cells, the activation of the canonical NF-κB pathway enhances the production and secretion of LPA, which activates the PLCε–PKD axis in an autocrine manner and leads to activation and cytoplasmic localization of RSK. The activated RSK, instead of IKK, phosphorylates IκB in the cytoplasm and induces its degradation, thereby activating NF-κB in a late time course [13]. To gain an insight into the molecular mechanisms of the PLCε‘s action, we examined the effects of the inhibitors of IKK and PKD on Cxcl5 expression in cultured *PLCε*^+/+^ AECs in 16 h after the LPS stimulation, which corresponded to the late phase of NF-κB activation. As a result, the LPS-induced elevation of the *Cxcl5* mRNA level was almost completely abolished by the treatment with the IKK inhibitor while it was substantially suppressed by the treatment with the PKD inhibitor (Fig. 6a). Also, the nuclear translocation of the p65 subunit of NF-κB observed in *PLCε*^+/+^ AECs was largely suppressed in *PLCε*^*∆X/∆X*^ AECs in 90 min after LPS stimulation, which also corresponded to the late phase of NF-κB activation (Fig. 6b). Likewise, the LPS-induced increase in the phosphorylation of PKD and IκB in *PLCε*^+/+^ AECs was substantially suppressed in *PLCε*^*∆X/∆X*^ AECs (Fig. 6c). These results indicated that PLCε plays a crucial role in LPS-induced NF-κB activation via activation of the PLCε-PKD-IκB-NF-κB pathway, thereby augmenting the expression of the neutrophil-attracting chemokine Cxcl5.

## Discussion

We demonstrated that PLCε plays a crucial role in the development of LPS-induced experimental ALI. By employing a similar experimental ALI model, Bijli et al. had reported a crucial role of PLCε in mediating endothelial cell inflammation and barrier disruption [26]. In the present study, we focused on the role of PLCε in AECs, which had been shown to act as the main source of pro-inflammatory cytokines and chemokines and thereby play a key role in the pathogenesis and resolution of ALI through regulation of neutrophil influx [27]. Also, AECs are known to play a pivotal role in innate immunity associated with the expression of TLR2 and TLR4 [28, 29]. TLR4 is a major receptor of LPS, and the LPS/TLR4 signaling is relayed by activation of the MyD88-dependent and MyD88-independent pathways, inducing the mRNA expression of pro-inflammatory cytokines and type I interferon [30, 31].

Our results using the lung tissues and primary AEC cultures from *PLCε*^+/+^ and *PLCε*^Δ*X/ΔX*^ mice suggested that PLCε might facilitate LPS-induced neutrophilic inflammation through augmentation of the production of the CXC family of chemokines, in particular Cxcl5, from AECs. Among the LPS-induced proinflammatory cytokines, we focused our study on chemokines that attract and activate neutrophils, in particular the CXC chemokines possessing the CXC cysteine motif [32]. The CXC chemokines are divided into two subgroups depending on the presence or absence of the Glu-Leu-Arg (ELR) motif. The CXC chemokines with the ELR motif, exemplified by Cxcl1, Cxcl2, Cxcl5 and Cxcl15, induce neutrophil chemotaxis, and Cxcl1, Cxcl2, Cxcl5 and Cxcl15 had been reported to function as important neutrophil chemo-attractants associated with lung inflammation in mice [32, 33]. Among them, we focused on Cxcl5 because it is produced mainly by AECs and because it is known to induce neutrophil trafficking in response to LPS exposure in the lung [24]. A key role of Cxcl5 had been shown in lung inflammation such as that occurred in the mouse model of pneumococcal pneumonia [34, 35]. Moreover, it had been reported that neutralization of Cxcl5 in the lung suppressed neutrophilic inflammation in a mouse model of LPS-induced ALI [6]. Furthermore, it had been observed that the levels of Cxcl5 in BALF of patients with ARDS were high [36–38]. These findings strongly supported our notion that the PLCε-mediated production of Cxcl5 from AECs might be causal to the pathogenesis of the LPS-induced ALI.

It is known that the expression of CXC chemokines including Cxcl5 is regulated by NF-κB [39, 40]. The expression of Cxcl5 accompanying pneumococcal pneumonia had been shown to be induced by NF-κB activation [33]. Here we showed that PLCε augments LPS-induced Cxcl5 expression through NF-κB activation via the PLCε-PKD-IκB pathway in AECs. This pathway had been demonstrated to be responsible for the late phase of NF-κB activation upon stimulation of cultured human colon epithelial cells by TNF-α, while the early phase was mediated by the canonical pathway using IKK for IκB phosphorylation and linked to the late phase by LPA [13]. In AECs, it is likely that the link between the early and late phases is provided by S1P, which was known to induce PLCε activation [9] and cooperate with the TLR4 signaling to enhance cytokine production in human gingival epithelial cells [41] through engagement of its GPCR receptors. The importance of the PLCε-PKD-IκB pathway in mediating NF-κB activation downstream of TLRs had been suggested by the observations that PKD was essential for the MyD88-dependent TLR signaling [42] and that PKD was involved in chemokine release induced by TLR2, TLR4 and TLR5 [43]. In our present study, the evidence for the critical role of the PLCε-PKD-IκB pathway came from the observation that the LPS-induced Cxcl5 expression was suppressed by the inhibitors of IKK and PKD and that the LPS-induced nuclear translocation of NF-κB and phosphorylation of PKD and IκB were suppressed in *PLCε*^*∆X/∆X*^ AECs. The IKK inhibitor was expected to suppress both the early and late phases of NF-κB activation while the PKD inhibitor was expected to suppress only the late phase. Our present observation that the PKD inhibitors resulted in substantial inhibition of LPS-induced Cxcl5 expression suggested that the PLCε-PKD-IκB pathway played a major role in the late phase NF-κB activation.

## Conclusions

The results, taken together, indicated that PLCε plays a crucial role in neutrophilic inflammation accompanying LPS-induced experimental ALI through augmentation of Cxcl5 production in AECs via activation of the PLCε-PKD-IκB-NF-κB pathway. This suggested that PLCε might become a promising candidate target for the treatment of ARDS, which was also supported by the fact that *PLCε*^*∆X/∆X*^ mice were born and grew normally except for the development of mild abnormality in the heart valves [44] predicting the low toxicity of PLCε-specific inhibitors.

## Additional files

Additional file 1: **Figure S1.** Primary cultured AECs with immunohistochemical staining. **Figure S2.** Effect of PLC*ε* on expression of chemokines and cytokines in LPS-induced ALI mice (related to Fig. 3). **Figure S3A.** Comparison of the expression levels of LPS-induced chemokine and cytokine(related to Fig. 5). **Figure S3B.** Role of PLCε in LPS-induced chemokine and cytokine production (related to Fig. 5). (PPTX 1631 kb)

Additional file 2: **Table S1.** List of primer sequences. (DOCX 21 kb)

## Abbreviations

AEC: Alveolar epithelial cell
ALI: Acute lung injury
ARDS: Acute respiratory distress syndrome
BALF: Bronchoalveolar lavage fluid
CCL: Chemokine C-C motif ligand
CXCL: Chemokine C-X-C motif ligand
DAG: Diacylglycerol
ELR: Glu-Leu-Arg
GPCR: G protein-coupled receptor
H&E: Hematoxylin and eosin
IKK: Inhibitor κB kinase
IκB: Inhibitor κB
LPA: Lysophosphatidic acid
LPS: Lipopolysaccharide
MyD88: Myeloid differentiation primary response gene 88
NF-κB: Nuclear factor-κB
PBS: Phosphate- buffered saline
PKD: Protein kinase D
PLC: Phospholipase C
pro-SPC: Prosurfactant protein C
qRT-PCR: Quantitative reverse transcription-polymerase chain reaction
S1P: Sphingosine-1-phosphate
SD: Standard deviation
TLR: Toll-like receptor
