# Supplementary material for: Phospholipase Cε plays a crucial role in neutrophilic inflammation accompanying acute lung injury through augmentation of CXC chemokine production from alveolar epithelial cells
Source: Respir Res. 2019 Jan 11;20:9. doi: 10.1186/s12931-019-0975-4 (PMC6330467; doi:10.1186/s12931-019-0975-4)
Supplement: Supplementary file 1 — Figure S1. Primary cultured AECs with immunohistochemical staining. Figure S2. Effect of PLCε on expression of chemokines and cytokines in LPS-induced ALI mice (related to Fig. 3). Figure S3A. Comparison of the expression levels of LPS-induced chemokine and cytokine(related to Fig. 5). Figure S3B. Role of PLCε in LPS-induced chemokine and cytokine production (related to Fig. 5). (PPTX 1631 kb) [file 12931_2019_975_MOESM1_ESM.pptx]

## Slide 1
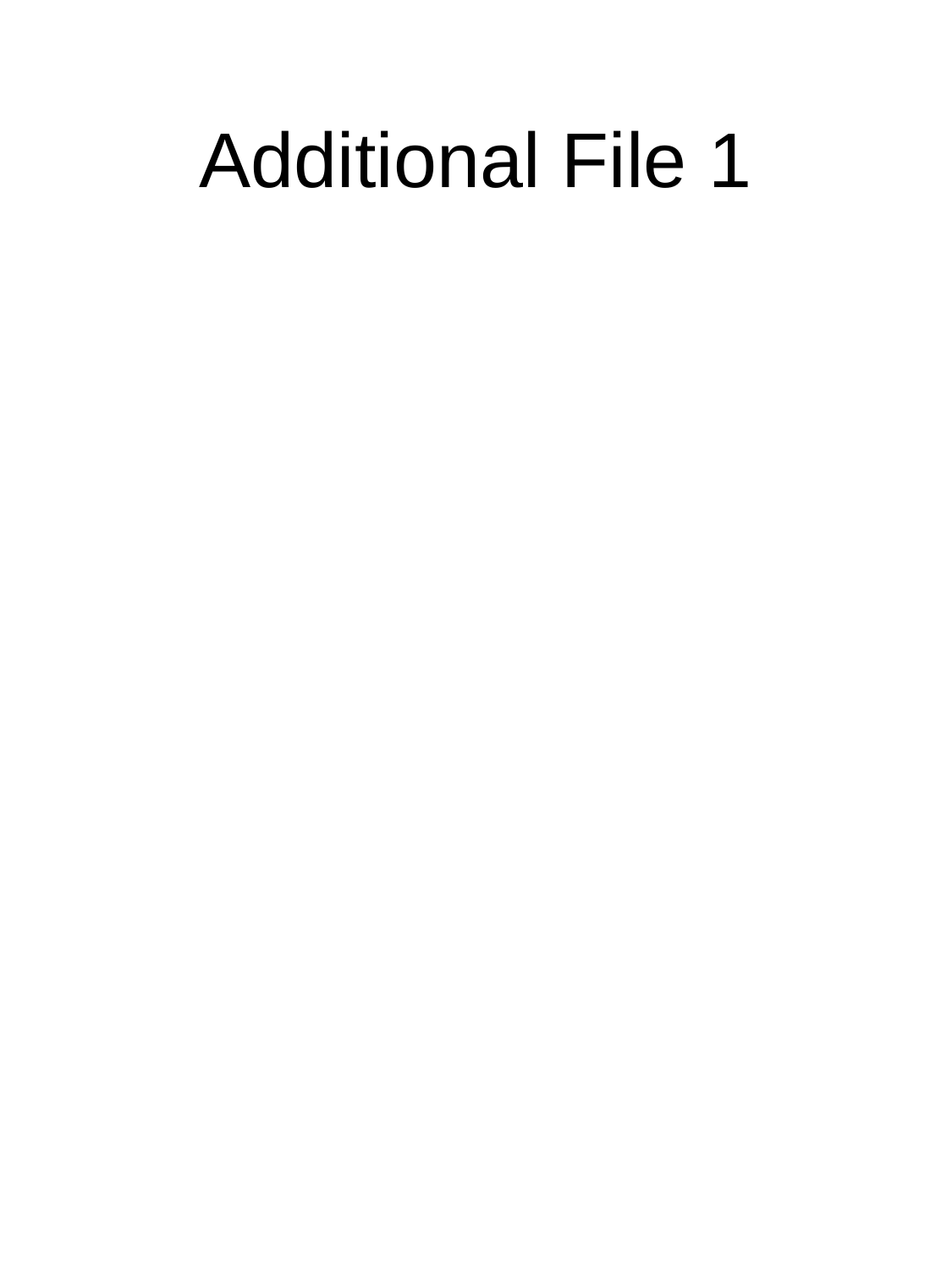

# Additional File 1

## Slide 2
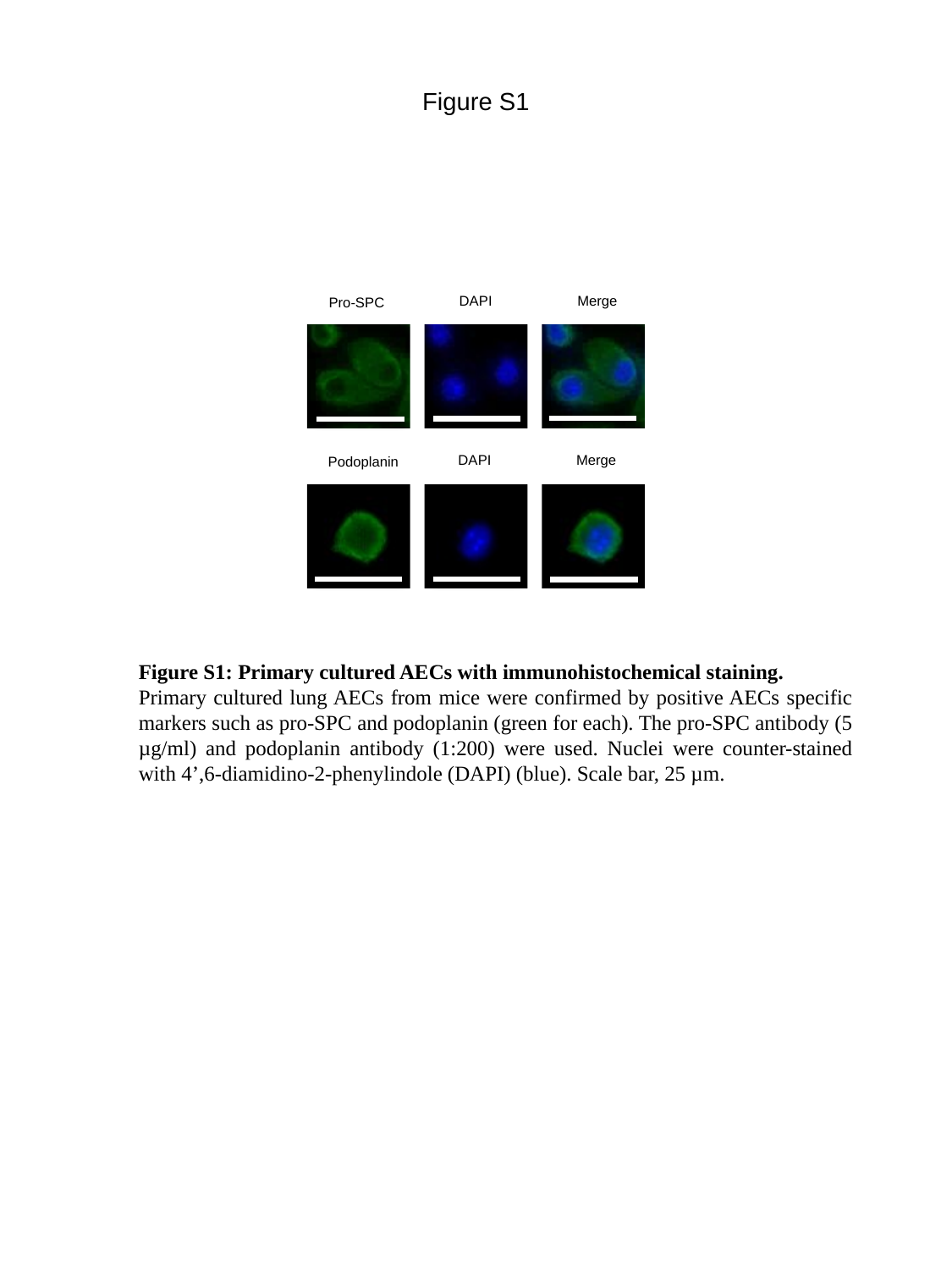

# Figure S1
DAPI
Merge
Pro-SPC
DAPI
Merge
Podoplanin
Figure S1: Primary cultured AECs with immunohistochemical staining.
Primary cultured lung AECs from mice were confirmed by positive AECs specific markers such as pro-SPC and podoplanin (green for each). The pro-SPC antibody (5 µg/ml) and podoplanin antibody (1:200) were used. Nuclei were counter-stained with 4’,6-diamidino-2-phenylindole (DAPI) (blue). Scale bar, 25 µm.

## Slide 3
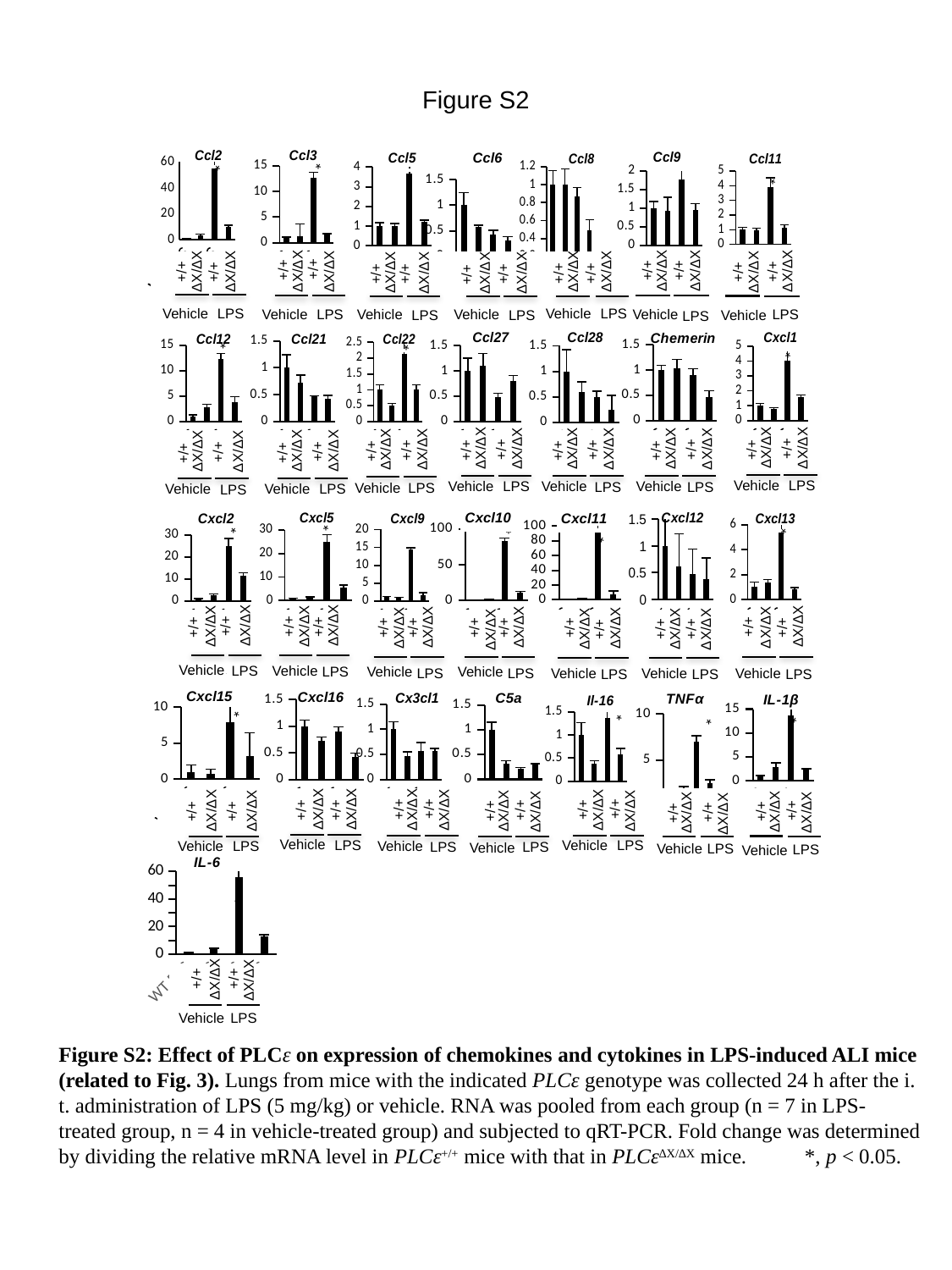

# Figure S2
### Chart: Ccl2
| Category | |
|---|---|
| WT PBS | 1.0 |
| KO PBS | 3.547173 |
| WT LPS | 56.75477 |
| KO LPS | 10.11439 |
### Chart: Ccl5
| Category | |
|---|---|
| WT PBS | 1.0 |
| KO PBS | 0.9794198 |
| WT LPS | 3.680753 |
| KO LPS | 1.222641 |
### Chart: Ccl8
| Category | |
|---|---|
| WT PBS | 1.0 |
| KO PBS | 1.00463 |
| WT LPS | 0.8665346 |
| KO LPS | 0.4919779 |
### Chart: Ccl11
| Category | |
|---|---|
| WT PBS | 1.0 |
| KO PBS | 0.9614826 |
| WT LPS | 3.890617 |
| KO LPS | 1.156687 |
### Chart: Ccl9
| Category | |
|---|---|
| WT PBS | 1.0 |
| KO PBS | 0.9351927 |
| WT LPS | 1.757265 |
| KO LPS | 0.9373552 |
### Chart: Ccl3
| Category | |
|---|---|
| WT PBS | 1.0 |
| KO PBS | 1.350351 |
| WT LPS | 12.72857 |
| KO LPS | 1.554731 |
### Chart: Ccl6
| Category | |
|---|---|
| WT PBS | 1.0 |
| KO PBS | 0.5703821 |
| WT LPS | 0.4352756 |
| KO LPS | 0.3070759 |*
*
*
*
+/+
+/+
+/+
+/+
ΔX/ΔX
ΔX/ΔX
+/+
+/+
ΔX/ΔX
+/+
ΔX/ΔX
ΔX/ΔX
ΔX/ΔX
+/+
ΔX/ΔX
ΔX/ΔX
ΔX/ΔX
ΔX/ΔX
+/+
+/+
ΔX/ΔX
+/+
ΔX/ΔX
+/+
ΔX/ΔX
+/+
ΔX/ΔX
+/+
*
Vehicle
Vehicle
LPS
LPS
Vehicle
LPS
Vehicle
Vehicle
Vehicle
LPS
LPS
Vehicle
LPS
LPS
### Chart: Chemerin
| Category | |
|---|---|
| WT PBS | 1.0 |
| KO PBS | 1.030492 |
| WT LPS | 0.9054247 |
| KO LPS | 0.4579732 |
### Chart: Ccl22
| Category | |
|---|---|
| WT PBS | 1.0 |
| KO PBS | 0.4931162 |
| WT LPS | 2.133662999999999 |
| KO LPS | 1.012789 |
### Chart: Ccl27
| Category | |
|---|---|
| WT PBS | 1.0 |
| KO PBS | 1.099363 |
| WT LPS | 0.4741233 |
| KO LPS | 0.80107 |
### Chart: Ccl28
| Category | |
|---|---|
| WT PBS | 1.0 |
| KO PBS | 0.6001239 |
| WT LPS | 0.4919786 |
| KO LPS | 0.2511578 |
### Chart: Ccl21
| Category | |
|---|---|
| WT PBS | 1.0 |
| KO PBS | 0.7269858 |
| WT LPS | 0.4611581 |
| KO LPS | 0.4175439 |
### Chart: Cxcl1
| Category | |
|---|---|
| WT PBS | 1.0 |
| KO PBS | 0.7508861 |
| WT LPS | 4.032469 |
| KO LPS | 1.598442 |
### Chart: Ccl12
| Category | |
|---|---|
| WT PBS | 1.0 |
| KO PBS | 2.808892999999998 |
| WT LPS | 12.29501 |
| KO LPS | 3.689265 |*
*
*
ΔX/ΔX
+/+
ΔX/ΔX
ΔX/ΔX
+/+
ΔX/ΔX
ΔX/ΔX
+/+
ΔX/ΔX
+/+
+/+
ΔX/ΔX
ΔX/ΔX
ΔX/ΔX
+/+
+/+
ΔX/ΔX
+/+
+/+
+/+
ΔX/ΔX
+/+
ΔX/ΔX
ΔX/ΔX
+/+
ΔX/ΔX
+/+
+/+
Vehicle
LPS
Vehicle
Vehicle
Vehicle
LPS
LPS
LPS
LPS
Vehicle
Vehicle
Vehicle
LPS
LPS
### Chart: Cxcl12
| Category | |
|---|---|
| WT PBS | 1.0 |
| KO PBS | 0.6099102 |
| WT LPS | 0.467597 |
| KO LPS | 0.3868915 |
### Chart: Cxcl10
| Category | |
|---|---|
| WT PBS | 1.0 |
| KO PBS | 1.677911 |
| WT LPS | 82.90199 |
| KO LPS | 11.73976 |
### Chart: Cxcl13
| Category | |
|---|---|
| WT PBS | 1.0 |
| KO PBS | 1.372364 |
| WT LPS | 5.364078999999972 |
| KO LPS | 0.8293182 |
### Chart: Cxcl9
| Category | |
|---|---|
| WT PBS | 1.0 |
| KO PBS | 0.8274038 |
| WT LPS | 14.28733 |
| KO LPS | 1.572796 |
### Chart: Cxcl11
| Category | |
|---|---|
| WT PBS | 1.0 |
| KO PBS | 1.981603 |
| WT LPS | 94.13552 |
| KO LPS | 7.110741 |
### Chart: Cxcl5
| Category | |
|---|---|
| WT PBS | 1.0 |
| KO PBS | 1.397965 |
| WT LPS | 24.99088 |
| KO LPS | 5.69618699999997 |
### Chart: Cxcl2
| Category | |
|---|---|
| WT PBS | 1.0 |
| KO PBS | 2.770214 |
| WT LPS | 24.99088 |
| KO LPS | 11.36609 |*
*
*
*
*
+/+
ΔX/ΔX
+/+
+/+
ΔX/ΔX
+/+
ΔX/ΔX
+/+
+/+
ΔX/ΔX
ΔX/ΔX
+/+
+/+
+/+
+/+
+/+
ΔX/ΔX
ΔX/ΔX
ΔX/ΔX
+/+
+/+
ΔX/ΔX
+/+
ΔX/ΔX
ΔX/ΔX
ΔX/ΔX
ΔX/ΔX
ΔX/ΔX
+/+
+/+
Vehicle
Vehicle
LPS
Vehicle
Vehicle
LPS
Vehicle
LPS
LPS
Vehicle
Vehicle
LPS
LPS
LPS
### Chart: Il-16
| Category | |
|---|---|
| WT PBS | 1.0 |
| KO PBS | 0.3833316 |
| WT LPS | 1.372368 |
| KO LPS | 0.577011 |
### Chart: IL-1β
| Category | |
|---|---|
| WT PBS | 1.0 |
| KO PBS | 2.757454 |
| WT LPS | 13.6422 |
| KO LPS | 2.361988 |
### Chart: Cx3cl1
| Category | |
|---|---|
| WT PBS | 1.0 |
| KO PBS | 0.4697619 |
| WT LPS | 0.5664421 |
| KO LPS | 0.5625293 |
### Chart: Cxcl16
| Category | |
|---|---|
| WT PBS | 1.0 |
| KO PBS | 0.7337352 |
| WT LPS | 0.9054247 |
| KO LPS | 0.4243517 |
### Chart: C5a
| Category | |
|---|---|
| WT PBS | 1.0 |
| KO PBS | 0.3215985 |
| WT LPS | 0.2054224 |
| KO LPS | 0.2891722 |
### Chart: Cxcl15
| Category | |
|---|---|
| WT-PBS | 1.0 |
| KO-PBS | 0.7054746 |
| WT-LPS | 7.94476 |
| KO-LPS | 3.234038 |
### Chart: TNFα
| Category | |
|---|---|
| WT-PBS | 1.0 |
| KO-PBS | 1.82345 |
| WT-LPS | 6.980523 |
| KO-LPS | 2.485157 |*
*
*
*
+/+
+/+
+/+
+/+
+/+
ΔX/ΔX
ΔX/ΔX
+/+
+/+
+/+
ΔX/ΔX
ΔX/ΔX
ΔX/ΔX
+/+
+/+
+/+
ΔX/ΔX
+/+
ΔX/ΔX
ΔX/ΔX
ΔX/ΔX
+/+
ΔX/ΔX
+/+
ΔX/ΔX
ΔX/ΔX
ΔX/ΔX
ΔX/ΔX
### Chart: IL-6
| Category | |
|---|---|
| WT PBS | 1.0 |
| KO PBS | 4.093505 |
| WT LPS | 55.71543 |
| KO LPS | 13.05625 |Vehicle
LPS
Vehicle
LPS
Vehicle
Vehicle
LPS
LPS
LPS
Vehicle
Vehicle
LPS
LPS
Vehicle
*
+/+
+/+
ΔX/ΔX
ΔX/ΔX
Vehicle
LPS
Figure S2: Effect of PLCε on expression of chemokines and cytokines in LPS-induced ALI mice (related to Fig. 3). Lungs from mice with the indicated PLCε genotype was collected 24 h after the i. t. administration of LPS (5 mg/kg) or vehicle. RNA was pooled from each group (n = 7 in LPS-treated group, n = 4 in vehicle-treated group) and subjected to qRT-PCR. Fold change was determined by dividing the relative mRNA level in PLCε+/+ mice with that in PLCε∆X/∆X mice. *, p < 0.05.

## Slide 4
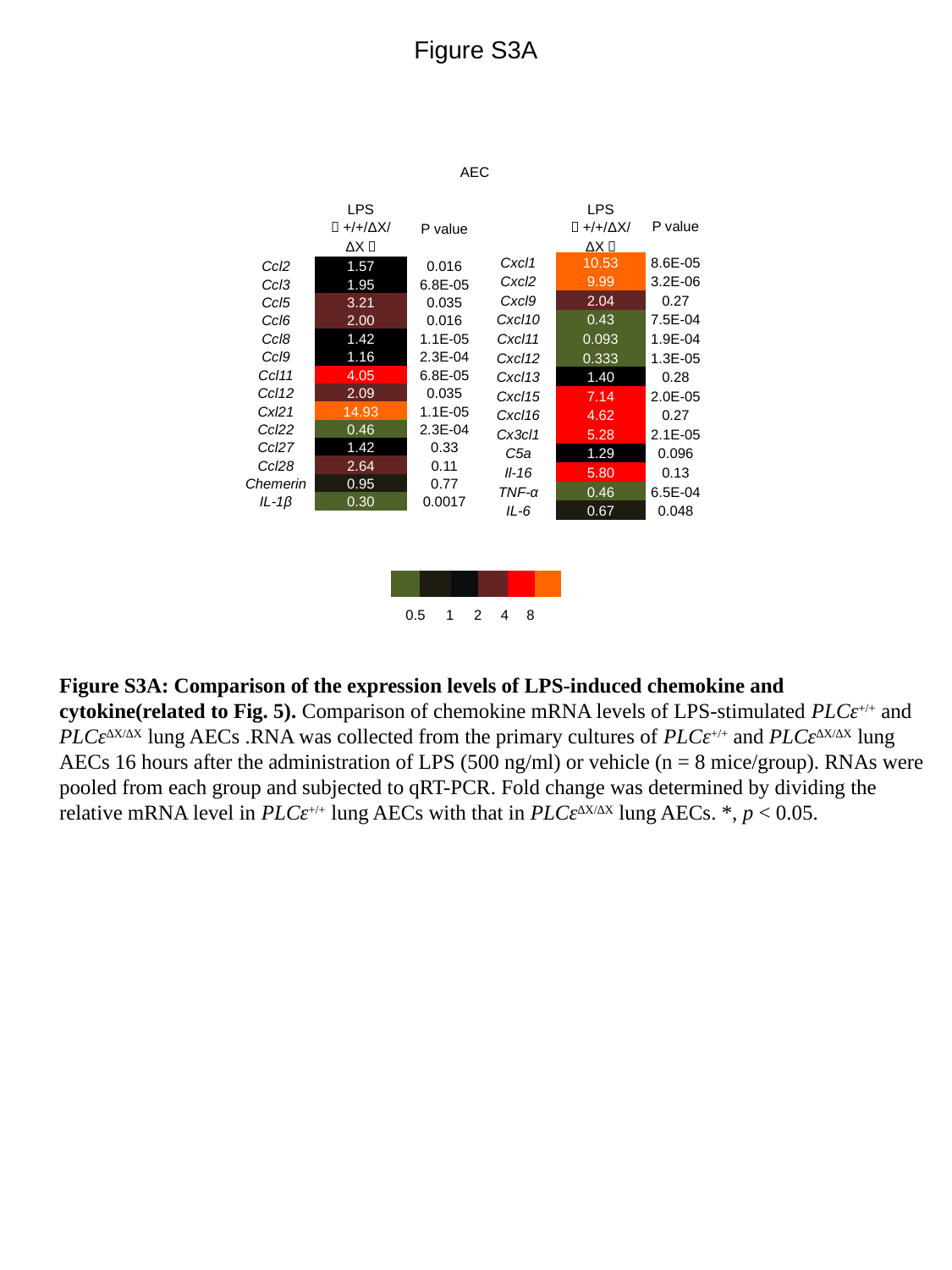

# Figure S3A
AEC
| | LPS （+/+/ΔX/ΔX） | P value |
| --- | --- | --- |
| Ccl2 | 1.57 | 0.016 |
| Ccl3 | 1.95 | 6.8E-05 |
| Ccl5 | 3.21 | 0.035 |
| Ccl6 | 2.00 | 0.016 |
| Ccl8 | 1.42 | 1.1E-05 |
| Ccl9 | 1.16 | 2.3E-04 |
| Ccl11 | 4.05 | 6.8E-05 |
| Ccl12 | 2.09 | 0.035 |
| Cxl21 | 14.93 | 1.1E-05 |
| Ccl22 | 0.46 | 2.3E-04 |
| Ccl27 | 1.42 | 0.33 |
| Ccl28 | 2.64 | 0.11 |
| Chemerin | 0.95 | 0.77 |
| IL-1β | 0.30 | 0.0017 |
| | LPS （+/+/ΔX/ΔX） | P value |
| --- | --- | --- |
| Cxcl1 | 10.53 | 8.6E-05 |
| Cxcl2 | 9.99 | 3.2E-06 |
| Cxcl9 | 2.04 | 0.27 |
| Cxcl10 | 0.43 | 7.5E-04 |
| Cxcl11 | 0.093 | 1.9E-04 |
| Cxcl12 | 0.333 | 1.3E-05 |
| Cxcl13 | 1.40 | 0.28 |
| Cxcl15 | 7.14 | 2.0E-05 |
| Cxcl16 | 4.62 | 0.27 |
| Cx3cl1 | 5.28 | 2.1E-05 |
| C5a | 1.29 | 0.096 |
| Il-16 | 5.80 | 0.13 |
| TNF-α | 0.46 | 6.5E-04 |
| IL-6 | 0.67 | 0.048 |
| | | | | | |
| --- | --- | --- | --- | --- | --- |
0.5
1
2
8
4
Figure S3A: Comparison of the expression levels of LPS-induced chemokine and cytokine(related to Fig. 5). Comparison of chemokine mRNA levels of LPS-stimulated PLCε+/+ and PLCε∆X/∆X lung AECs .RNA was collected from the primary cultures of PLCε+/+ and PLCε∆X/∆X lung AECs 16 hours after the administration of LPS (500 ng/ml) or vehicle (n = 8 mice/group). RNAs were pooled from each group and subjected to qRT-PCR. Fold change was determined by dividing the relative mRNA level in PLCε+/+ lung AECs with that in PLCε∆X/∆X lung AECs. *, p < 0.05.

## Slide 5
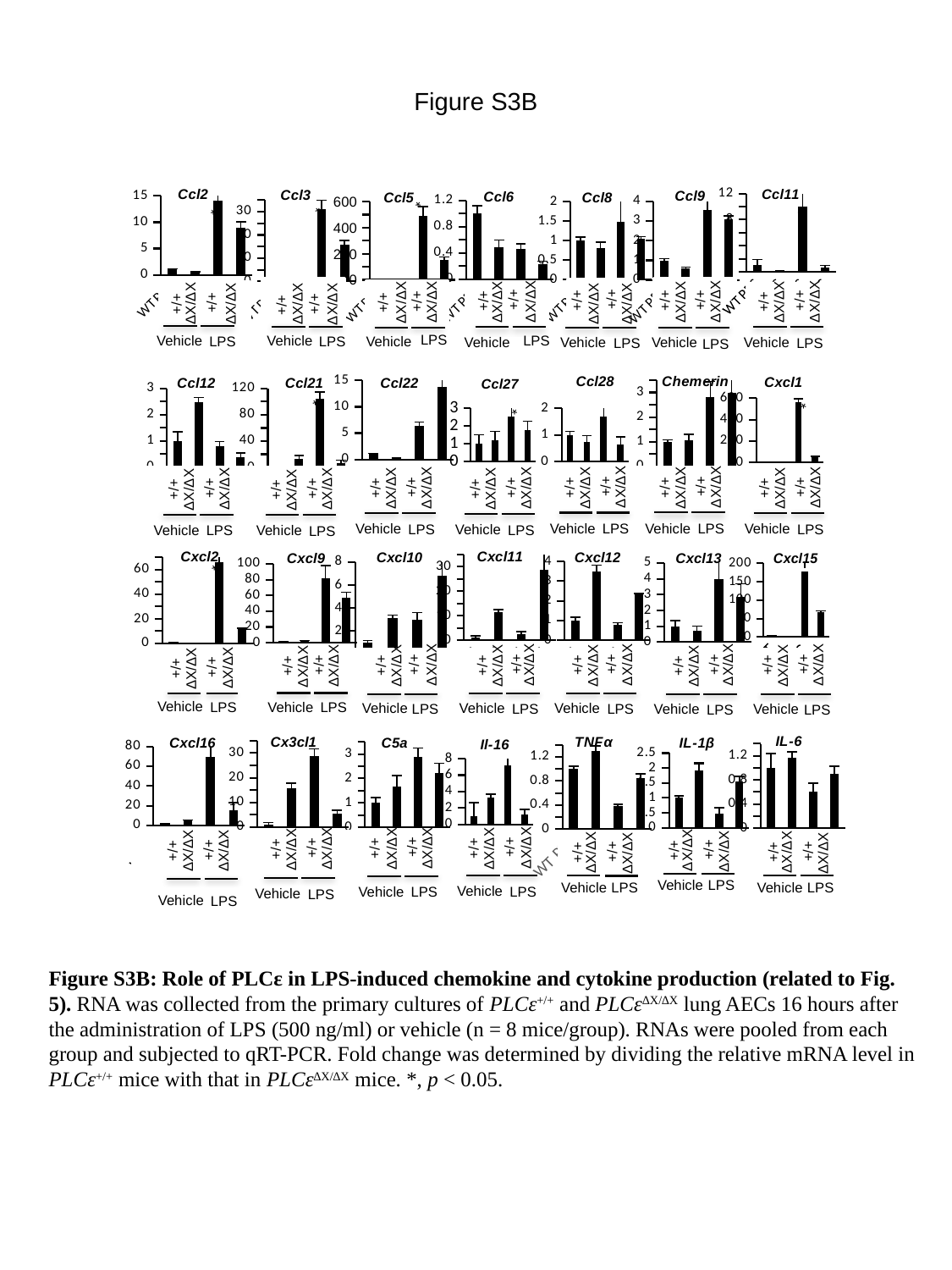

# Figure S3B
### Chart: Ccl6
| Category | |
|---|---|
| WT PBS | 1.0 |
| KO PBS | 0.4863272 |
| WT LPS | 0.4579719 |
| KO LPS | 0.2284577 |
### Chart: Ccl5
| Category | |
|---|---|
| WT PBS | 1.0 |
| KO PBS | 2.406045999999953 |
| WT LPS | 487.7513999999989 |
| KO LPS | 151.8671 |
### Chart: Ccl9
| Category | |
|---|---|
| WT PBS | 1.0 |
| KO PBS | 0.6113206 |
| WT LPS | 3.563596 |
| KO LPS | 3.066660999999999 |
### Chart: Ccl11
| Category | |
|---|---|
| WT PBS | 1.0 |
| KO PBS | 0.09539112 |
| WT LPS | 10.05611 |
| KO LPS | 0.6705106 |
### Chart: Ccl8
| Category | |
|---|---|
| WT PBS | 1.0 |
| KO PBS | 0.8160136 |
| WT LPS | 1.481098 |
| KO LPS | 1.044877 |
### Chart: Ccl2
| Category | |
|---|---|
| WT PBS | 1.0 |
| KO PBS | 0.5823642 |
| WT LPS | 14.09064 |
| KO LPS | 8.979681000000001 |
### Chart: Ccl3
| Category | |
|---|---|
| WT PBS | 1.0 |
| KO PBS | 0.6372813 |
| WT LPS | 31.05317 |
| KO LPS | 15.85282 |*
*
*
*
+/+
+/+
ΔX/ΔX
ΔX/ΔX
Vehicle
LPS
+/+
+/+
+/+
+/+
+/+
+/+
+/+
ΔX/ΔX
ΔX/ΔX
+/+
+/+
ΔX/ΔX
ΔX/ΔX
ΔX/ΔX
ΔX/ΔX
+/+
+/+
ΔX/ΔX
ΔX/ΔX
ΔX/ΔX
ΔX/ΔX
ΔX/ΔX
+/+
ΔX/ΔX
LPS
Vehicle
LPS
Vehicle
Vehicle
LPS
LPS
Vehicle
Vehicle
Vehicle
LPS
LPS
### Chart: Chemerin
| Category | |
|---|---|
| WT PBS | 1.0 |
| KO PBS | 1.079227 |
| WT LPS | 2.828427 |
| KO LPS | 2.989694 |
### Chart: Ccl22
| Category | |
|---|---|
| WT PBS | 1.0 |
| KO PBS | 0.17883 |
| WT LPS | 6.305724 |
| KO LPS | 13.80066 |
### Chart: Ccl12
| Category | |
|---|---|
| WT PBS | 1.0 |
| KO PBS | 2.473685 |
| WT LPS | 0.806641 |
| KO LPS | 0.385998 |
### Chart: Ccl21
| Category | |
|---|---|
| WT PBS | 1.0 |
| KO PBS | 13.4233 |
| WT LPS | 104.6915 |
| KO LPS | 7.012862 |
### Chart: Cxcl1
| Category | |
|---|---|
| WT PBS | 1.0 |
| KO PBS | 0.3415102 |
| WT LPS | 564.1759 |
| KO LPS | 53.56927 |
### Chart: Ccl28
| Category | |
|---|---|
| WT PBS | 1.0 |
| KO PBS | 0.7286668 |
| WT LPS | 1.685682 |
| KO LPS | 0.6402328 |
### Chart: Ccl27
| Category | |
|---|---|
| WT PBS | 1.0 |
| KO PBS | 1.19196 |
| WT LPS | 2.50823 |
| KO LPS | 1.761335 |*
*
+/+
+/+
ΔX/ΔX
ΔX/ΔX
Vehicle
LPS
+/+
+/+
ΔX/ΔX
ΔX/ΔX
Vehicle
LPS
+/+
+/+
ΔX/ΔX
ΔX/ΔX
Vehicle
LPS
+/+
+/+
ΔX/ΔX
ΔX/ΔX
Vehicle
LPS
+/+
+/+
ΔX/ΔX
ΔX/ΔX
Vehicle
LPS
+/+
+/+
ΔX/ΔX
ΔX/ΔX
Vehicle
LPS
+/+
+/+
ΔX/ΔX
ΔX/ΔX
Vehicle
LPS
ΔX/ΔX
+/+
ΔX/ΔX
ΔX/ΔX
ΔX/ΔX
ΔX/ΔX
+/+
+/+
+/+
+/+
ΔX/ΔX
ΔX/ΔX
+/+
+/+
ΔX/ΔX
+/+
+/+
+/+
### Chart: Cxcl12
| Category | |
|---|---|
| WT PBS | 1.0 |
| KO PBS | 3.482209999999998 |
| WT LPS | 0.7845849 |
| KO LPS | 2.351102 |
### Chart: Cxcl11
| Category | |
|---|---|
| WT PBS | 1.0 |
| KO PBS | 11.60491 |
| WT LPS | 2.669678 |
| KO LPS | 28.83991 |
### Chart: Cxcl10
| Category | |
|---|---|
| WT PBS | 1.0 |
| KO PBS | 3.09512 |
| WT LPS | 2.941727 |
| KO LPS | 6.821063 |
### Chart: Cxcl13
| Category | |
|---|---|
| WT PBS | 1.0 |
| KO PBS | 0.6845971 |
| WT LPS | 3.981549999999999 |
| KO LPS | 2.841523 |
### Chart: Cxcl9
| Category | |
|---|---|
| WT PBS | 1.0 |
| KO PBS | 2.175987 |
| WT LPS | 81.85504999999995 |
| KO LPS | 56.82024000000001 |
### Chart: Cxcl2
| Category | |
|---|---|
| WT PBS | 1.0 |
| KO PBS | 0.2049487 |
| WT LPS | 66.10416 |
| KO LPS | 11.65872 |
### Chart: Cxcl15
| Category | |
|---|---|
| WT PBS | 1.0 |
| KO PBS | 0.1615444 |
| WT LPS | 176.8849 |
| KO LPS | 67.96246 |*
*
+/+
+/+
ΔX/ΔX
ΔX/ΔX
Vehicle
LPS
+/+
ΔX/ΔX
+/+
ΔX/ΔX
Vehicle
LPS
+/+
+/+
ΔX/ΔX
ΔX/ΔX
Vehicle
LPS
+/+
+/+
ΔX/ΔX
ΔX/ΔX
Vehicle
LPS
+/+
+/+
ΔX/ΔX
ΔX/ΔX
Vehicle
LPS
+/+
+/+
ΔX/ΔX
ΔX/ΔX
Vehicle
LPS
+/+
+/+
ΔX/ΔX
ΔX/ΔX
Vehicle
LPS
### Chart: Cxcl16
| Category | |
|---|---|
| WT PBS | 1.0 |
| KO PBS | 5.19936 |
| WT LPS | 69.7925 |
| KO LPS | 15.11946 |
### Chart: Cx3cl1
| Category | |
|---|---|
| WT PBS | 1.0 |
| KO PBS | 15.54452 |
| WT LPS | 28.80671999999998 |
| KO LPS | 5.457847 |
### Chart: C5a
| Category | |
|---|---|
| WT PBS | 1.0 |
| KO PBS | 1.677909 |
| WT LPS | 2.894534 |
| KO LPS | 2.239741 |
### Chart: Il-16
| Category | |
|---|---|
| WT PBS | 1.0 |
| KO PBS | 3.275384 |
| WT LPS | 7.185036999999975 |
| KO LPS | 1.238276 |+/+
+/+
ΔX/ΔX
ΔX/ΔX
+/+
+/+
ΔX/ΔX
ΔX/ΔX
Vehicle
LPS
+/+
+/+
ΔX/ΔX
ΔX/ΔX
Vehicle
LPS
+/+
+/+
ΔX/ΔX
ΔX/ΔX
Vehicle
LPS
*
Vehicle
LPS
### Chart: IL-6
| Category | |
|---|---|
| WT PBS | 1.0 |
| KO PBS | 1.167429 |
| WT LPS | 0.6070994 |
| KO LPS | 0.907522 |
### Chart: TNFα
| Category | |
|---|---|
| WT PBS | 1.0 |
| KO PBS | 1.292355 |
| WT LPS | 0.3842195 |
| KO LPS | 0.8408975 |
### Chart: IL-1β
| Category | |
|---|---|
| WT PBS | 1.0 |
| KO PBS | 1.905279 |
| WT LPS | 0.4746721 |
| KO LPS | 1.561938 |+/+
+/+
ΔX/ΔX
ΔX/ΔX
Vehicle
LPS
+/+
+/+
ΔX/ΔX
ΔX/ΔX
Vehicle
LPS
+/+
+/+
ΔX/ΔX
ΔX/ΔX
Vehicle
LPS
Figure S3B: Role of PLCε in LPS-induced chemokine and cytokine production (related to Fig. 5). RNA was collected from the primary cultures of PLCε+/+ and PLCε∆X/∆X lung AECs 16 hours after the administration of LPS (500 ng/ml) or vehicle (n = 8 mice/group). RNAs were pooled from each group and subjected to qRT-PCR. Fold change was determined by dividing the relative mRNA level in PLCε+/+ mice with that in PLCε∆X/∆X mice. *, p < 0.05.
