# Supplementary material for: Phospholipase Cε plays a crucial role in neutrophilic inflammation accompanying acute lung injury through augmentation of CXC chemokine production from alveolar epithelial cells
Source: Respir Res. 2019 Jan 11;20:9. doi: 10.1186/s12931-019-0975-4 (PMC6330467; doi:10.1186/s12931-019-0975-4)
Supplement: Supplementary file 2 — Table S1. List of primer sequences. (DOCX 21 kb) [file 12931_2019_975_MOESM2_ESM.docx]

**Supplemental Table**

**e-Table S1. List of primer sequences**

| Gene | Forward Primer(5’-3’) | Reverse Primer(5’-3’) | References |
| --- | --- | --- | --- |
| *Ccl11* | CAAGACCAAACTGGCCAAGG | GAATCCTGCACCCACTTCTTCT | 1 |
| *Ccl12* | GGGAAGCTGTGATCTTCAGG | GGGAACTTCAGGGGGAAATA | 2 |
| *Ccl2* | TCCCAATGAGTAGGCTGGAG | TCTGGACCCATTCCTTCTTG | 3 |
| *Ccl21* | TCCCGGCAATCCTGTTCTT | CCTTCCTCAGGGTTTGCACA | 4 |
| *Ccl22* | AAGACAGTATCTGCTGCCAGG | GATCGGCACAGATATCTCGG | 5 |
| *Ccl27* | ATAGACAGCCACTCCCAAGC | ACAGTCCCTTGGAGCCTTTT | 6 |
| *Ccl28* | TGGCAAAAGCCACATTCATA | CATGCCAGAGTCGGACAGAA | 6 |
| *Ccl3* | ATCATGAAGGTCTCCACCAC | TCTCAGGCATTCAGTTCCAG | 7 |
| *Ccl5* | TGGCTCGGACACCACTCCCTG | CTCCTTGACGTGGGCACGAGG | 8 |
| *Ccl6* | GGCTTTGGAATGTGTCTGGT | CTGGCCCCGTAGTTCTATGA | 6 |
| *Ccl8* | AGCCCAGGCACCATCTGCTTGTAA | TGCCCCATGGAAGCTGTGGTTTTC | 9 |
| *Ccl9* | AGTGGTCTGTGGGACTTTGG | CAGACCTGTGGCTGCATAGA | 6 |
| *Chemerin* | ACCTGTGCAGTTGGCCTTCC | GAGGATCCTGAGGCCCTTGCT | 10 |
| *C5a* | AGGGTACTTTGCCTGCTGAA | TGTGAAGGTGCTCTTGGATG | 11 |
| *Cx3cl1* | GGAAAGAAACGTGGTCCAGA | GCCCTCAGAATCACAGGGTA | 6 |
| *Cxcl1* | GCACCCAAACCGAAGTCATAG | AGAAGCCAGCGTTCACCAGA | 12 |
| *Cxcl10* | GCCGTCATTTTCTGCCTCAT | GCTTCCCTATGGCCCTCATT | 6 |
| *Cxcl11* | AGTAACGGCTGCGACAAAGT | GCATGTTCCAAGACAGCAGA | 6 |
| *Cxcl12* | GCTCTGCATCAGTGACGGTA | TAATTTCGGGTCAATGCACA | 13 |
| *Cxcl13* | CATCATGAGGTGGTGCAAAG | GGGTCACAGTGCAAAGGAAT | 6 |
| *Cxcl16* | GGGAAGAGTTTTCACCACCA | GGTTGGGTGTGCTCTTTGTT | 6 |
| *Cxcl2* | GCCCAGACAGAAGTCATAGCC | CTCCTCCTTTCCAGGTCAGTTA | 12 |
| *Cxcl9* | AAAATTTCATCACGCCCTTG | TCTCCAGCTTGGTGAGGTCT | 6 |
| *Il-16* | CAGCCATTCAGCCTACACCA | CGTCCTCCATCTTGCTTTCC | 12 |
| *Cxcl5* | CTGCCCCTTCCTCAGTCATA | GTGCATTCCGCTTAGCTTTC | 14 |
| *Cxcl15* | CGTCCCTGTGACACTCAAGA | TAATTGGGCCAACAGTAGCC | 14 |
| *TNF-α* | GAGTGACAAGCCTGTAGCC | CTCCTGGTATGAGATAGCAAA | 12 |
| *IL-1β* | TGGGAAACAACAGTGGTCAGG | CCATCAGAGGCAAGGAGGAA | 12 |
| *IL-6* | CCTCTGGTCTTCTGGAGTACC | ACTCCTTCTGTGACTCCAGC | 15 |

CCL, CC motif chemokine ligand; CX3CL, chemokine C-X3-C motif ligand: CXCL, chemokine C-X-C motif ligand; IL, interleukin; TNF, tumor necrosis factor.

1. Chou DL, Daugherty BL, McKenna EK, Hsu WM, Tyler NK, Plopper CG, et al. Chronic aeroallergen during infancy enhances eotaxin-3 expression in airway epithelium and nerves. Am J Respir Cell Mol Biol. 2005; 33: 1-8.
2. Sautter NB, Shick EH, Ransohoff RM, Charo IF, Hirose K. CC chemokine receptor 2 is protective against noise-induced hair cell death: studies in CX3CR1(+/GFP) mice. J Assoc Res Otolaryngol. 2006;7:361-372.
3. Ha J, Choi HS, Lee Y, Kwon HJ, Song YW, Kim HH. CXC chemokine ligand 2 induced by receptor activator of NF-kappa B ligand enhances osteoclastogenesis. J Immunol. 2010;184:4717-4724.
4. Afshar R, Strassner JP, Seung E, Causton B, Cho JL, Harris RS, et al. Compartmentalized chemokine-dependent regulatory T-cell inhibition of allergic pulmonary inflammation. J Allergy Clin Immunol. 2013;131:1644-1652.
5. Su SB, Grajewski RS, Luger D, Agarwal RK, Silver PB, Tang J, et al. Altered chemokine profile associated with exacerbated autoimmune pathology under conditions of genetic interferon-gamma deficiency. Invest Ophthalmol Vis Sci. 2007;48:4616-4625.
6. Lionakis MS, Fischer BG, Lim JK, Swamydas M, Wan W, Richard Lee CC, et al. Chemokine receptor Ccr1 drives neutrophil-mediated kidney immunopathology and mortality in invasive candidiasis. PLoS pathog. 2012;8:e1002865.
7. Yang X, Lu P, Fujii C, Nakamoto Y, Gao JL, Kaneko S, et al. Essential contribution of a chemokine, CCL3, and its receptor, CCR1, to hepatocellular carcinoma progression. Int J Cancer. 2006;118:1869-1876.
8. Zhang C, Li Y, Wu Y, Wang L, Wang X, Du J. Interleukin-6/signal transducer and activator of transcription 3 (STAT3) pathway is essential for macrophage infiltration and myoblast proliferation during muscle regeneration. J Biol Chem. 2013;288:1489-1499.
9. Di Valentin E, Crahay C, Garbacki N, Hennuy B, Guéders M, Noël A, et al. New asthma biomarkers: lessons from murine models of acute and chronic asthma. Am J Physiol Lung Cell Mol Physiol. 2009;296:L185-197.
10. Stein S, Lohmann C, Handschin C, Stenfeldt E, Borén J, Lüscher TF, et al. ApoE-/- PGC-1α-/- mice display reduced IL-18 levels and do not develop enhanced atherosclerosis. PLoS One. 2010;5:e13539.
11. Li X, Ding F, Zhang X, Li B, Ding J. The Expression Profile of Complement Components in Podocytes. Int J Mol Sci. 2016;17:471.
12. Sun B, Hu X, Liu G, Ma B, Xu Y, Yang T, et al. Phosphatase Wip1 negatively regulates neutrophil migration and inflammation. J Immunol. 2004;192:1184-1195.
13. Yoon KA, Chae YM, Cho JY. FGF2 stimulates SDF-1 expression through the Erm transcription factor in Sertoli cells. J Cell Physiol. 2009;220:245-256.
14. Ma B, Kang MJ, Lee CG, Chapoval S, Liu W, Chen Q, et al. Role of CCR5 in IFN-γ-induced and cigaratte smoke-induced emphysema. J Clin Invest. 2005;115:3460-3472.
15. Liu T, Shi Y, Du J, Ge X, Teng X, Liu L, et al. Vitamin D treatment attenuates 2,4,6-trinitrobenzene sulphonic acid (TNBS)-induced colitis but not oxazolone-induced colitis. Scientific reports. 2016;1-10.
